# 2526 E-learning for best practices in social and behavioral research: A multisite pilot evaluation

**DOI:** 10.1017/cts.2018.206

**Published:** 2018-11-21

**Authors:** Susan L. Murphy, Elias M. Samuels, Christine Byks-Jazayeri, Ellen Champagne, Jordan Hahn, Brenda Eakin, Robert Kolb, Linda S. Behar-Horenstein, Susan Gardner, Fanny Ennever, Mary-Tara Roth, Margarita L. Dubocovich

**Affiliations:** 1 University of Michigan School of Medicine; 2 University of Florida; 3 Boston Medical Center and Boston University Medical Campus; 4 State University of New York

## Abstract

OBJECTIVES/SPECIFIC AIMS: To evaluate the NIH-sponsored Best Practices for Social and Behavioral Research e-learning course. METHODS/STUDY POPULATION: Four universities partnered in a pilot study to evaluate this new course. Outcomes from 294 participants completing the course included efficient progress through the training, perceived relevance of the course to current work, level of engagement with the course material, intent to work differently as a result of the course, and downloading digital resources. RESULTS/ANTICIPATED RESULTS: Participants rated the course as relevant and engaging (6.4 and 5.8 on a 7-point Likert scale) and 96% of respondents said they would recommend the course to colleagues. Qualitative analysis of participant testimonials suggested that most respondents had a readiness to change in the way they worked as a result of the course. Overall, results suggest participants completed the course efficiently, perceived outcomes positively and worked differently after the training. DISCUSSION/SIGNIFICANCE OF IMPACT: These results will inform new guidelines for future participants (e.g., average time to complete, expectations for knowledge checks in the training). Future studies should include larger samples and closer coordination and communication between study sites.

